# Artificial DnaJ Protein for protein production and conformational diseases

**DOI:** 10.1038/s41598-017-09067-7

**Published:** 2017-08-17

**Authors:** Akinori Hishiya, Keizo Koya

**Affiliations:** Sola Biosciences, Inc., 27 Strathmore Road, Natick, MA 01760 USA

## Abstract

For secreted proteins, proper protein folding is essential not only for biological function but also for secretion itself. Proteins with folding problems are trapped in the endoplasmic reticulum (ER) and are eventually degraded in the cytoplasm. In this study, we exploited co-expression of an artificial fusion protein, based on the sequence of a DnaJ protein, which could interact as co-chaperones in the Hsp70-based protein-folding system, with target recombinant secreted proteins to enhance their production and secretion. The J-domain sequence or a fragment thereof was conjugated to a target protein–binding domain that was capable of binding to a portion of the target-protein sequence. Production of many of the target proteins was significantly upregulated when co-expressed with the J-domain fusion protein. Surprisingly, the enhancement of secretion was observed even when the J-domain had a mutation in the HPD motif, which is necessary for J-protein–Hsp70 interactions, suggesting the phenomenon observed is independent on functional J-protein–Hsp70 interactions. This technology has great potential for not only enhancing the production of recombinant proteins, but also to treat conformational diseases such as cystic fibrosis, and Alpha-1 antitrypsin deficiency.

## Introduction

When proteins like secreted or membrane proteins are expressed in eukaryotic cells, the partly translated peptide synthesized from its mRNA is harnessed to the endoplasmic reticulum (ER) with the aid of signal-recognition particles (SRPs), and translation resumes on the ER surface^1^. The synthesized peptide is then released into the ER lumen, followed by protein folding and modifications such as glycosylation and the formation of disulfide bonds^2^. A strict quality control system exists in the ER to survey whether the synthesized proteins have adopted their proper conformations and whether or not the modifications are adequate^3, 4^. Cells possess a set of proteins that serve as molecular chaperones to aid in the folding and refolding of nascent and mature proteins into their proper conformations^5–7^. For instance, misfolded or unfolded proteins are detected by ER chaperones, such as Bip protein, a heat shock 70 kDa ER-resident protein, and another round of protein folding is attempted^8^. Synthesized proteins are loaded into or onto a transport vesicle only when they are deemed acceptable by this strict cellular quality-control system.

Besides the simple effect of reduced secretion, misfolded proteins can also give rise to more deleterious effects. In the ER, misfolded or unfolded proteins can yield a stress signal (ER stress) that brings about many cellular events, termed the unfolded protein response (UPR), such as the suppression of *de novo* translation in order to diminish the burden on the ER. In addition, ER stress also induces the expression of a set of proteins that help to upregulate the proteins involved in ER protein folding^9^. However, when the ER stress exceeds a certain cellular threshold, the cell death response is normally initiated (apoptosis)^10, 11^.

Hence, the overall quality control system in the ER has a significant impact on the production of expressed proteins^12^. Many attempts have been made to improve protein folding and to modify the cellular quality control system to foster greater expression, though these approaches have tended to act in a general way on all secreted proteins made by the host cell with unwanted cellular effects that limit their efficiency and applicability.

We attempted to discover a protein domain or subdomain that had the potential to improve secretion of expressed proteins. In order to engineer it for a response specific to a target protein, we first conjugated these domains to a target protein (IL13Rα2) that would be easily degraded because of a protein-folding problem. Through screening, we identified a set of protein subdomains from the DnaJ protein family, involved within the Hsp70 machinery as a co-chaperone. The J-domain sequence or a fragment thereof was conjugated to a target protein-binding domain that was capable of binding to a portion of the target-protein sequence. The resultant J-domain fusion protein was expressed with the target protein in the mammalian cell model, HEK-293 human embryonic kidney cells. We next tested this system on a range of therapeutically useful model target proteins, such as IL13Rα2 (IL13Rα2TF-Fc), TNFRSF1B (TNFRSF1BTF-Fc), and TNFRSF1A (TNFRSF1ATF-Fc), and a human monoclonal antibody, anti-IL8, by assessing the expression and activity of the target proteins produced. The experiment of truncation and mutation of J-domain sequence showed that protein secretion of the target proteins was enhanced by the J-domain fusion protein independently of the functional J-protein–Hsp70 interactions. We also exploited the system for a folding-deficient allele of Alpha-1 antitrypsin (Z mutant) to establish if this artificial DnaJ protein system had any potential for treating a conformational disease, such as Alpha-1 antitrypsin deficiency.

## Results

### Screening for a protein domain with protein expression-enhancing activity

To search for a protein sequence with protein expression-enhancing activity, we isolated cDNAs of a domain or part of a domain (subdomain) from various chaperone proteins and co-chaperone proteins and conjugated the cDNAs to the human *IL13Rα2* gene to express various IL13Rα2 fusion proteins. IL13Rα2 was employed as a target protein in this screening because IL13Rα2 is known to be unstable based on a protein-folding problem.

**Figure 1:**
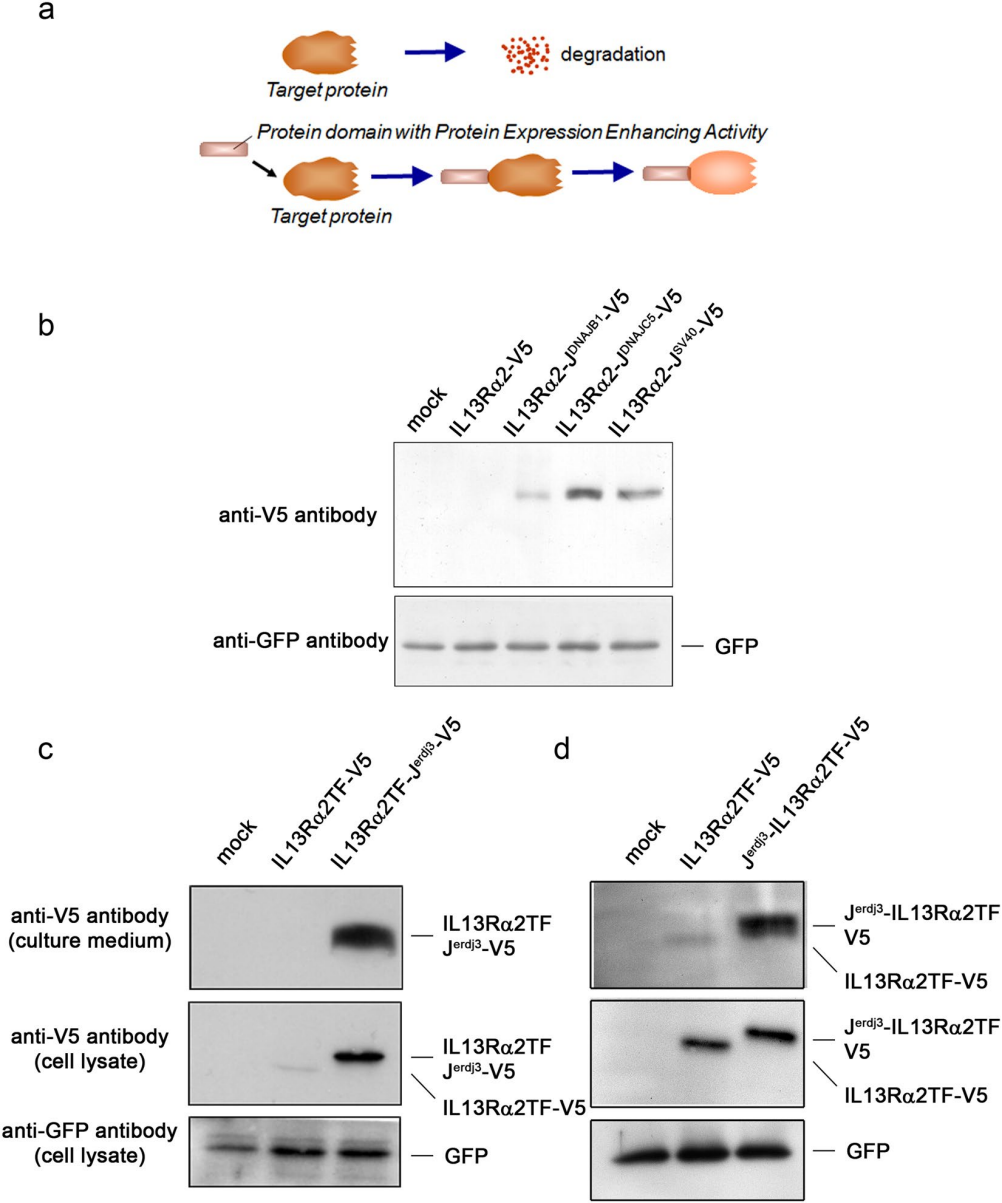
Screening for a protein domain/subdomain that controls protein folding/cellular quality system. (**a**) Illustrates screening for a sequence to improve the stability of a protein of interest by incorporation of protein domains or subdomains from various chaperone or co-chaperone proteins. (**b**) Shows X-ray film images of chemiluminescent signals of a western blot (immunoblot) analysis of cell lysates, indicating relative levels of the expression of V5-tagged IL13Rα2 wild-type (IL13Rα2-V5) protein or fusion protein counterparts (IL13Rα2-J-domain fragment-V5) in transfected cells. HEK293 cells were transfected with an empty vector (lane 1); the plasmid for IL13Rα2 alone (lane 2); fusion protein of IL13Rα2 with a J-domain sequence derived from DNAJB1 (J^DNAJB1^; lane 3); CSP (J^DNAJC5^; lane 4); and SV40 large T antigen (J^SV40^; lane 5). All transfectants were co-transfected with a reporter plasmid expressing green fluorescent protein (GFP) to demonstrate the successful transfection and operability of the transfectants (lower panel). (**c**) HEK293 cells were transfected with an empty vector (lane 1); a plasmid for a V5-tagged truncated form (“TF”) of IL13Rα2 receptor protein (IL13Rα2TF-V5; lane 2); the extracellular portion of the IL13Rα2 receptor protein containing the ligand binding domain; and a fusion protein of IL13Rα2TF with a J-domain fragment sequence from Erdj3 (IL13Rα2TF -J^erdj3^-V5; lane 3). Two days later, the cell culture medium (right panel) and cell lysate (left panel) were harvested, and western blot analysis was conducted with an anti-V5 antibody. A reporter plasmid (GFP) was co-transfected to monitor transfection efficiency (lower panel). (**d**) J-domain sequence was inserted between signal sequence and a V5-tagged IL13Rα2TF and expressed in HEK293 cells. After the expression, the culture medium (top panel) and cell lysate (middle panel) were harvested to analyze the expression of the protein. Lane1: mock transfectant, lane2: IL13Rα2TF-V5, and lane 3 shows the N-terminal fusion protein with the J-domain sequence (J^erdj3^-IL13Rα2TF-V5). GFP was co-transfected for transfection efficiency (lower panel).

Fusion proteins of IL13Rα2 with a domain from a chaperone or co-chaperone protein were expressed in HEK293 cells, and the levels of expressed V5-tagged IL13Rα2 (IL13Rα2-V5) were compared with that of various IL13Rα2 fusion proteins comprising a chaperone or co-chaperone domain in transfected HEK293 cells (Fig. 1a). Through the screening, a set of subdomains was identified. All of these subdomains were located in the J-domain of the DnaJ protein family. As shown in the second lane of Fig. 1b, only a faint band was detected for the V5-tagged IL13Rα2, indicating poor expression. However, the IL13Rα2 fusion protein with the subdomain from DNAJB1, a DnaJ family protein, was significantly stabilized (IL13Rα2-J^DNAJB1^-V5: lane 3). The corresponding subdomain from cysteine string protein (CSP; DNAJC5) also stabilized the V5-tagged IL13Rα2 (IL13Rα2-J^DNAJC5^-V5: lane 4), and interestingly, even a subdomain derived from the virus protein, SV40 large T antigen, stabilized the fused protein (IL13Rα2-J^SV40^-V5: lane 5).

There are two transcripts of IL13Rα2: the 1152-bp transcript encodes the full-length protein (utilized as the target in the aforementioned screen) while the 1020-bp transcript lacks exon10^13^. The short form of IL13Rα2 yields a soluble truncated form of IL13Rα2 (IL13Rα2TF), which lacks both the transmembrane domain and cytoplasmic tail of the longer form^13^. Of note is that the truncated form of IL13Rα2 has been employed as a decoy receptor to treat asthma by binding to IL13 molecules without transducing a signal to the cell to set off an inflammatory response^14, 15^. For such therapeutic applications, a genetically engineered, truncated form of IL13Rα2 has been expressed in mammalian cells and purified as a protein secreted into culture media.

Samples of cell lysates and culture media from two-day cultures of HEK293 cells transfected with plasmids containing either V5-tagged IL13Rα2TF or a fusion protein comprising V5-tagged IL13Rα2TF and the J-domain sequence from ERdj3 (DNAJB11), an ER-resident DnaJ protein, were analyzed for the expression of V5-tagged IL13Rα2TF or V5-tagged IL13Rα2TF fusion protein by immunoblot assay with an anti-V5 antibody. As shown in Fig. 1c, the expression of the V5-tagged IL13Rα2TF protein was barely detected in the media as well as in cell lysates (IL13Rα2TF-V5: lane 2). In contrast, the level of expression of the V5-tagged IL13Rα2TF fusion protein was dramatically higher inside the cell (cell lysate samples) and was secreted into the medium (IL13Rα2TF-J^erdj3^-V5: lane 3), suggesting that a J-domain sequence corresponding to that screened with full length IL13Rα2 enhances not only the stability of the fused proteins, but also their secretion when the J-domain sequence is conjugated to the secreted protein. A similar effect was observed when the J-domain sequence was attached to the N-terminus of the truncated form of IL13Rα2 (Fig. 1d).

### Protein expression enhanced by fusion protein with the identified J-domain sequence

**Figure 2:**
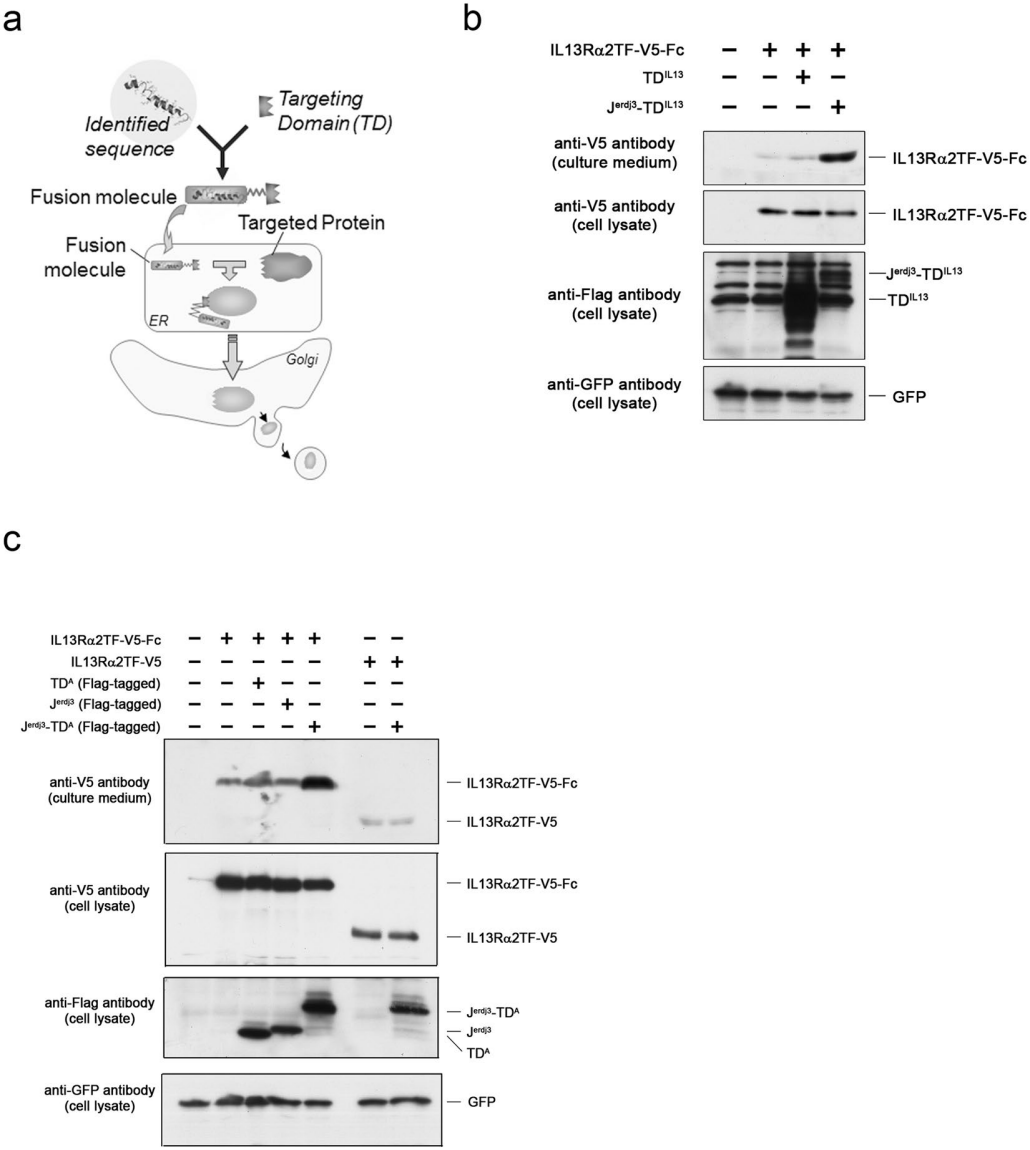
Protein expression enhanced by fusion protein with the identified J-domain sequence. (**a**) Depicts a diagram of a general scheme in which a fusion molecule of the J-domain fragment sequence enhances the expression of a target protein of interest. In the scheme, a J-domain fusion protein comprises a J-domain fragment sequence linked to a target protein-binding domain, wherein the target-binding domain specifically binds to a target protein of interest and the J-domain fragment sequence modulates the protein folding/cellular quality control system. This results in the enhanced secretion of a target protein. (**b**) HEK293 cells were transfected to express V5-tagged IL13Rα2TF (IL13Rα2TF-V5) with (+) or without (−) a Flag-tagged J-domain fusion protein incorporating the IL13 sequence as a targeting domain (J^erdj3^-TD^IL13^). IL13Rα2TF-Fc was also expressed with a Flag-tagged targeting domain (TD^IL13^). Two days later, the culture medium and cell lysates were harvested and assayed by western blot analysis, as indicated. In the absence of a J-domain fusion protein, most of IL13Rα2TF-V5 was retained inside the cells and not secreted (lane 2). The secretion of IL13Rα2TF-V5 was greatly enhanced by co-transfection with the J-domain fragment fusion protein (J^erdj3^-TD^IL13^; lane 4), but not by the targeting domain only (TD^IL13^; lane 3). The control (lane 1) indicates empty vector transfection control. All transfectants were co-transfected with a reporter plasmid expressing GFP. The cell lysate membrane was subsequently probed with an anti-Flag antibody (to detect Flag-tagged proteins), and anti-GFP antibody (as a transfection control) (bottom panel). (**c**) Either V5-tagged Fc fusion protein with IL13Rα2TF (IL13Rα2TF-V5-Fc; lanes 2 through 5) or V5-tagged IL13Rα2TF (IL13Rα2TF-V5; lanes 6 and 7) was expressed in HEK293 cells with (+) or without (−) a Flag-tagged J-domain fusion protein incorporating protein A sequence as a targeting domain (J^erdj3^-TD^A^; lanes 5 and 7). IL13Rα2TF-Fc was also expressed with either a targeting domain only (TD^A^; lane 3) or a Flag-tagged J-domain fragment sequence only (J^erdj3^; lane 4). Two days later, the culture medium and cell lysates were harvested, followed by western blot analysis to detect the expressed proteins. Neither a targeting domain–only nor a J-domain fragment–only sequence improved the secretion of IL13Rα2TF-V5-Fc (lanes 3 and 4, respectively). However, the secretion of IL13Rα2TF-V5-Fc was greatly enhanced by co-transfection with the J-domain fragment fusion protein (lane 5), while the secretion of IL13Rα2TF-V5 was not affected by the J-domain fragment fusion protein, which was designed to bind to Fc fusion protein (lane 7). The control (lane 1) indicates empty vector transfection control. The plasmid-carrying GFP gene was also transfected as transfection reporter. The cell lysate membrane was subsequently probed with an anti-Flag antibody (to detect Flag-tagged proteins), and anti-GFP antibody (as a transfection control) (bottom panel).

As noted previously, the identified J-domain fragment sequence enhanced the expression and secretion of the target protein when the sequence was conjugated to the target protein (Fig. 1). We exploited the identified J-domain fragment sequence further to develop the technology being described herein, in which the J-domain sequence is not fused to the target protein. With this technology, a fusion protein is instead created comprising the J-domain fragment sequence linked to a target protein-binding domain (targeting domain; TD) that has an affinity for and binds to the target protein of interest (Fig. 2a).

To investigate whether protein expression-enhancing activity is observed in this arrangement, IL13Rα2TF protein (target protein) was expressed in HEK293 cells with a fusion protein carrying a J-domain fragment sequence (effecter domain) and IL13 (targeting domain: TD) flanked by a flexible linker. When the IL13Rα2TF protein was expressed with IL13 (TD^IL13^; Fig. 2b, lane 3), the secretion of IL13Rα2TF protein was not improved. Interestingly, the fusion protein of the J-domain fragment significantly raised the level of secreted IL13Rα2TF protein expression (J^erdj3^-TD^IL13^; lane 4), more than the expression of IL13Rα2TF protein alone (lane 2) or with the co-expression of IL13Rα2TF protein and its IL13 ligand (lane 3). These results indicate that a J-domain fragment sequence is effective not only when it is conjugated to a target protein, but also when the target protein is expressed with a fusion protein composed of two structural domains, a J-domain fragment sequence and a targeting domain.

One format for designing therapeutically active fusion proteins combines a binding domain, which specifically binds a desired target molecule, linked to an immunoglobulin Fc region with an enhanced *in vivo* half-life^16^.

Bacteria and viruses have evolved means to escape the immune response of a host by expressing antibody-binding proteins, which bind to antibodies and neutralize their effects. Many such molecules have been discovered and utilized^17–19^. We took advantage of the combination of Fc fusion protein (target protein) and J-domain fragment fusion protein carrying Fc binding protein. We expressed V5-tagged IL13Rα2TF as a Fc-fusion (Fig. 2c, lanes 2–5) or non-Fc fusion protein (lane 6 and 7) in HEK293 cells with Fc domain–binding proteins fused with a J-domain fragment. When the Fc-fusion protein with IL13Rα2TF (IL13Rα2TF-Fc) was expressed in HEK293 cells, most of the Fc-fusion protein was retained in the host cell (lane 2). The fusion protein carrying Staphylococcal protein A (J^erdj3^-TD^A^) as a TD significantly enhanced the secretion of IL13Rα2TF-Fc fusion protein (Fig. 2c, lane 5) compared to IL13Rα2TF-Fc fusion protein expression alone (lane 2), although neither the targeting domain alone (TD^A^; lane 3) nor the J-domain fragment sequence alone (J^erdj3^; lane 4) enhanced the secretion of IL13Rα2TF-Fc protein.

Importantly, the J-domain fusion protein carrying protein A (J^erdj3^-TD^A^) did not upregulate either the intracellular protein levels or the secretion of the IL13Rα2TF, which was devoid of the Fc portion (lane 6 and 7). These results demonstrate the specificity of the artificial J-domain fusion protein in enhancing the secretion only for the intended target proteins.

### Engineered J-domain fusion protein for other target proteins

In order to address the question of whether enhanced production by an engineered J-domain fusion protein is widely observed, we tested the system with other target proteins. One of the most well-known Fc-fusion proteins is the therapeutic drug etanercept (commercially available as ENBREL), which possesses a truncated form of a tumor necrosis factor α receptor (TNFRSF1B) linked to an immunoglobulin Fc domain^20, 21^. Etanercept binds with and inhibits tumor necrosis factor (TNFα), which is a key immune system protein involved in a number of autoimmune diseases.

**Figure 3:**
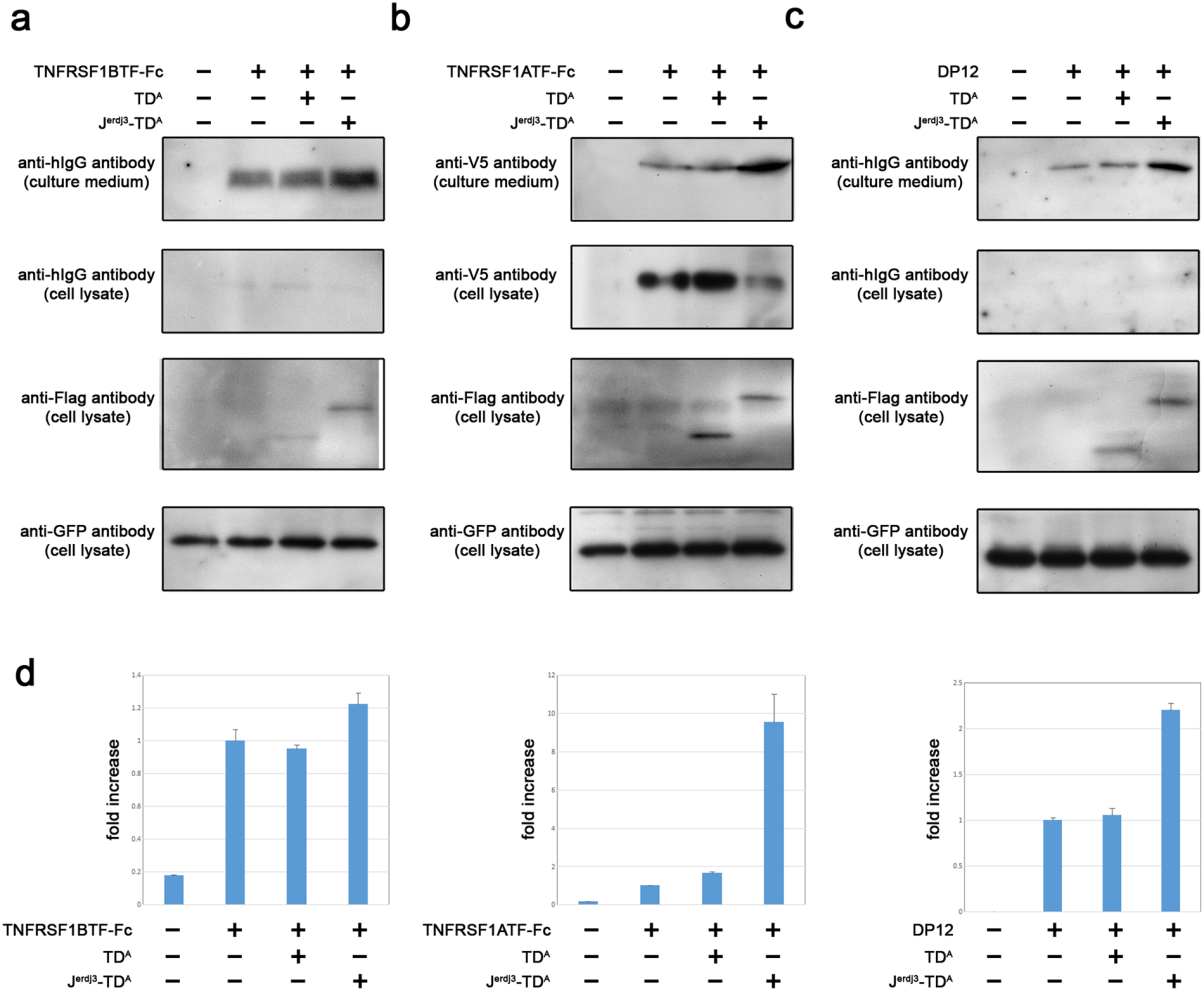
Enhanced secretion of Fc-fusion proteins and monoclonal antibodies. (**a**) Fc-fusion protein with the extracellular domain of Tumor necrosis factor receptor superfamily member 1B (TNFRSF1BTF-Fc) was expressed in HEK293 cells with (+) or without (−) a Flag-tagged protein A (TD^A^) or a Flag-tagged J-domain fragment fusion protein carrying protein A as a targeting domain (J^erdj3^-TD^A^). Two days later, the cell culture medium was harvested, and a western blot assay was performed with anti-human IgG antibody to detect TNFRSF1BTF-Fc. J^erdj3^-TD^A^ in cell lysate was detected by an anti-Flag antibody. GFP was expressed in all samples as a transfection reporter. (**b**) The same J-domain fragment fusion protein (J^erdj3^-TD^A^) was expressed with Fc-fusion protein of the V5-tagged Tumor necrosis factor receptor superfamily member 1 A (TNFRSF1A-V5-Fc) in HEK293 cells. TNFRSF1A-V5-Fc and J-domain fusion protein were detected in the cell culture medium and cell lysate, harvested two days after transfection, by western blot analysis using an anti-V5 antibody or anti-Flag antibody, respectively. A reporter plasmid (GFP) was co-transfected to monitor transfection efficiency (lower panel). (**c**) The dual-expression plasmid expressing both heavy chain and light chain for anti-IL8 antibody was transfected in HEK293 cells with (+) or without (−) a J-domain fragment fusion protein carrying protein A as a targeting domain (J^erdj3^-TD^A^). Two days later, the cell culture medium was harvested, and antibody production was detected by western blot analysis using an anti-human IgG antibody. The J-domain fragment fusion protein and reporter protein (GFP) was detected in the cell lysate by an anti-Flag antibody and anti-GFP antibody, respectively. (**d**) The culture media from cells expressing Fc fusion proteins or antibodies were harvested, and their antigen-binding activities were measured by ELISA. Fc fusion proteins and antibody were expressed with (forth bar) or without (second bar) targeting the J-domain fragment fusion proteins (J^erdj3^-TD^A^). As a control, target proteins were expressed with targeting domain only (TD^A^; third bar). The culture medium from control transfectant (empty vector transfection control) was shown in the left bar. Assay plates were coated with the corresponding antigens or binding ligands, and the secreted Fc fusion proteins or antibodies were applied to the respective plates. Bound antibodies or Fc fusion proteins were detected with HRP-conjugated anti-hIgG antibody.

We studied the expression of the Fc fusion protein with a truncated form of a TNFRSF1B (TNFRSF1BTF-Fc). The expression plasmids were transfected to HEK293 cells with or without a J-domain fragment fusion protein with protein A (J^erdj3^-TD^A^). Samples of cell lysate and culture medium from two-day cultures of transfected cells were analyzed using a western blot assay with an anti-human IgG antibody for expression of the TNFRSF1BTF-Fc protein. TNFRSF1BTF-Fc protein was well secreted and it was clearly detected in the culture media by western blot assay using anti-human IgG antibody, though only a faint band was observed in the cell lysate (Fig. 3a, lane 2), suggesting most of the expressed TNFRSF1BTF-Fc protein was efficiently secreted. The secretion of TNFR2TF-Fc protein was, however, enhanced when TNFRSF1BTF-Fc protein was expressed by the engineered J-domain fusion protein (lane 4). Importantly, expression of the targeting domain alone (TD^A^) did not change either the secretion or expression of TNFRSF1BTF-Fc (lane 3).

We attempted to apply the system to another type of tumor necrosis factor α receptor (TNFRSF1A). The V5-tagged fusion protein of the extracellular domain of TNFRSF1A (TNFRSF1ATF) with Fc domain (TNFRSF1ATF-V5-Fc) was expressed in human cells and its cell culture media were harvested to detect TNFRSF1ATF-V5-Fc. Contrary to TNFRSF1B, TNFRSF1ATF-V5-Fc was barely noticeable in the culture media, and most of the expressed proteins were in the cell lysate (Fig. 3b, lane 2). Interestingly, the production of TNFRSF1ATF-V5-Fc was significantly improved when TNFRSF1ATF-V5-Fc was expressed with the fusion protein of the J-domain fragment with protein A (J^erdj3^-TD^A^; lane 4). These results indicate that the expression system using the J-domain fusion protein enhances the secretion of Fc-fusion proteins, and the system works well when the target proteins are poorly secreted.

We examined whether the system also enhances the secretion of human monoclonal antibodies. Recombinant humanized anti-IL8 antibody was expressed in HEK293 cells and the culture medium was harvested to analyze the production of this antibody. Its expression was clearly detected by anti-human IgG antibody (Fig. 3c). Interestingly, more anti-IL8 antibody was observed in the culture medium when it was expressed with a J-domain fusion protein utilizing protein A as the targeting domain (J^erdj3^-TD^A^; lane 3).

The expressed proteins were further analyzed based on their epitope-binding ability. Purified recombinant TNFα was coated for Fc fusion proteins of TNFRSF1BTF or TNFRSF1ATF, followed by incubation with the cell culture medium. The Fc fusion proteins were detected using HRP-labeled anti-human IgG antibody (ELISA). When the coated TNFα was incubated with the cell culture medium from cells expressing Fc fusion proteins of TNFRSF1BTF or TNFRSF1ATF alone, a strong signal was detected (Fig. 3d, second bar in the first graph) compared with mock cultures from cells transfected with an expression vector lacking a structural gene for expressing any protein (no Fc fusion protein, first bar). As expected, when TNFα was incubated with a cell culture medium from cells expressing Fc fusion protein along with the J-domain fragment fusion protein carrying protein A, an additional signal was observed (J^erdj3^-TD^A^; fourth bar). Each sample (i.e., cell culture medium) was diluted and applied to calculate the fold increase (Y-axis).

As well as the affinity to TNFα, anti-IL8 antibody was analyzed with ELISA using purified recombinant IL8. The results of this series of experiments clearly showed that these Fc fusion proteins and the antibody, expressed at enhanced levels and secreted into the culture medium, retained their binding activity for their binding partners, indicating proper protein conformation (Fig. 3d).

### Engineered J-domain fusion protein with various targeting domains (TD)

J-domain fusion proteins incorporating protein A as the targeting domain proved effective for Fc-fusion proteins and antibodies (Figs 2 and 3). We examined whether other Fc-binding molecules were effective in this expression system. DnaJ fragment fusion proteins with various Fc-binding molecules were expressed with the Fc/IL13Rα2TF fusion protein (IL13Rα2TF-Fc) in HEK293 cells, and the presence of the fusion proteins in the culture media were analyzed by western blot.

**Figure 4:**
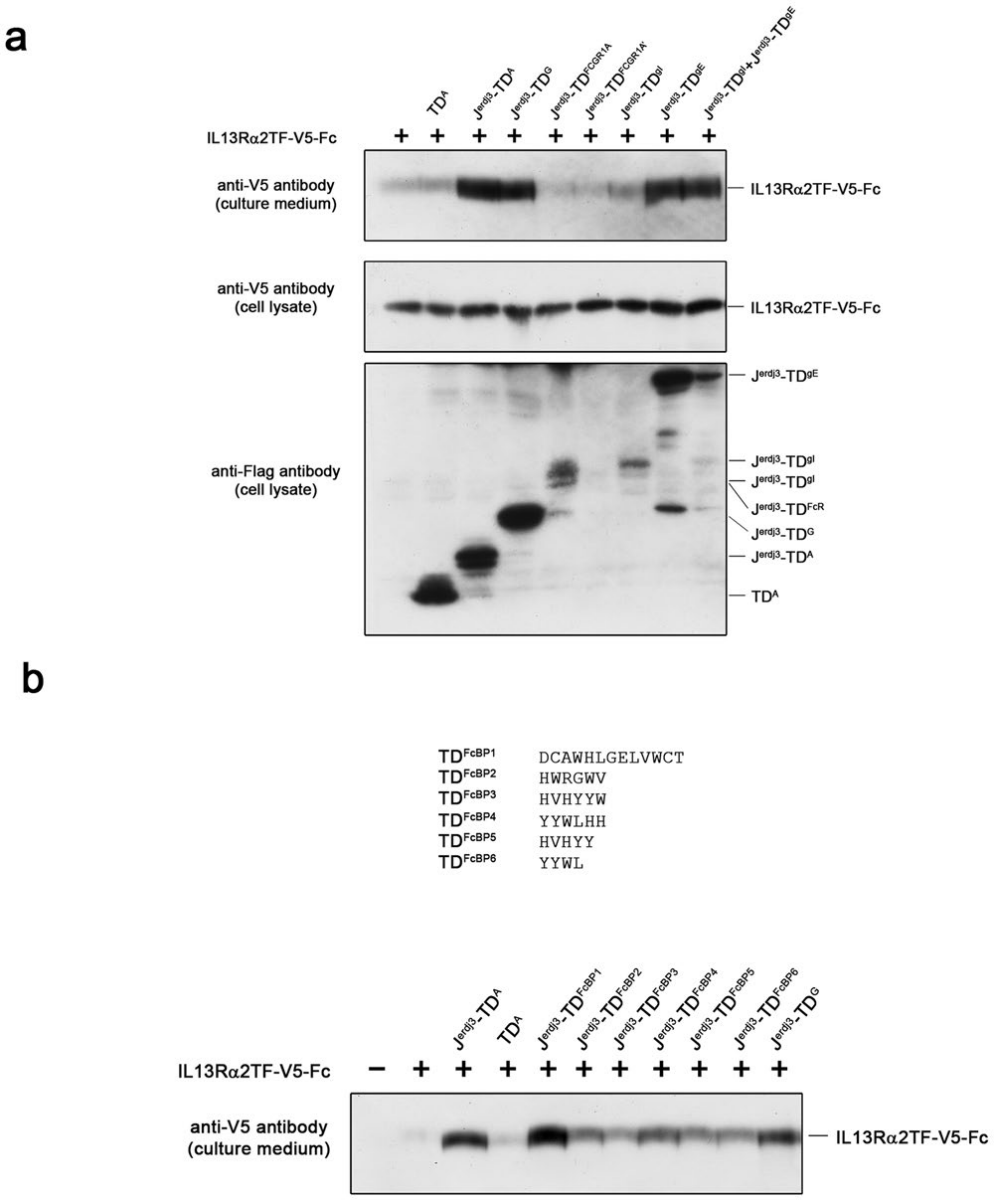
Targeting domain in J-domain fragment fusion protein. (**a**) V5-tagged IL13Rα2TF-Fc (IL13Rα2TF-V5-Fc) was expressed in HEK293 cells with (+) or without (−) a J-domain fragment fusion protein carrying protein A (J^erdj3^-TD^A^; lane 3), protein G (J^erdj3^-TD^G^; lane 4), FCGR1A (J^erdj3^-TD^FCGR1A^; lane 5), Macacine gammaherpesvirus 5 R1 (J^erdj3^-TD^FCGR1A’^; lane 6), glycoprotein I (J^erdj3^-TD^gI^; lane 7), glycoprotein E (J^erdj3^-TD^gE^; lane 8), and co-expression of gI and gE (J^erdj3^-TD^gI^/J^erdj3^-TD^gE^; lane 9). Two days later, the cell culture medium was harvested and the secreted protein was detected by western blot assay using an anti-V5 antibody. As a control, IL13Rα2TF-Fc was expressed alone (lane 1) or with a targeting domain only (TD^A^; lane 2). (**b**) The J-domain fragment fusion protein carrying various Fc-binding peptides (FcBPs) was expressed with V5-tagged IL13Rα2TF-Fc (IL13Rα2TF-V5-Fc) in HEK293 cells, and the secretion of the target protein (IL13Rα2TF-V5-Fc) was monitored by western blot assay using an anti-V5 antibody. Empty vector transfection control (lane 1), IL13Rα2TF-V5-Fc alone (lane 2), IL13Rα2TF-V5-Fc with a J-domain fusion protein carrying protein A (J^erdj3^-TD^A^; lane 3), FcBP1 (J^erdj3^-TD^FcBP1^; lane 5), FcBP2 (J^erdj3^-TD^FcBP2^; lane 6), FcBP3 (J^erdj3^-TD^FcBP3^; lane 7), FcBP4 (J^erdj3^-TD^FcBP4^; lane 8), FcBP5 (J^erdj3^-TD^FcBP5^; lane 9), FcBP6 (J^erdj3^-TD^FcBP6^; lane 10), and protein G (J^erdj3^-TD^G^; lane 11). IL13Rα2TF-V5-Fc was also expressed with protein A alone (TD^A^; lane 4).

Other microbial-derived proteins, such as *Streptococcus* sp. protein G (G), were also effective as a TD (Fig. 4a, lane 4), while human IgG Fc receptor I (FCGR1A) failed to exhibit an effect (lane 5). Macacine gammaherpesvirus 5 R1 was also tested since it has a partial homology with FCGR1A, though the expression could not be detected (FCGR1A’; lane 6). Glycoprotein E (gE), produced by human herpes virus type I, has also been reported to have an affinity for human antibodies in a complex with glycoprotein I (gI), and it was reported that gE has the ability to bind to the Fc portion in this complex^22, 23^. In fact, the fusion protein carrying gE showed the effect irrespective of its singular or hetero dimer status with gI (lane 8 and 9). However, gI itself does not possess antibody-binding ability, and the fusion proteins carrying gI did not demonstrate any activity (lane 7).

We also assessed a synthesized peptide that was discovered through the search for an Fc-binding peptide^24–26^. We incorporated six kinds of Fc-binding peptide (referred to herein as “FcBP1–6”) into a fusion protein and expressed them with IL13Rα2TF-Fc-fusion protein in HEK293 cells (Fig. 4b, lanes 5–10). Although all of these tested synthetic peptides successfully enhanced the secretion of IL13Rα2TF-Fc-fusion protein, FcBP1 had the most pronounced activity when incorporated into a fusion protein with the J-domain fragment sequence (lane 5).

### Identification of the essential amino acid sequence

**Figure 5:**
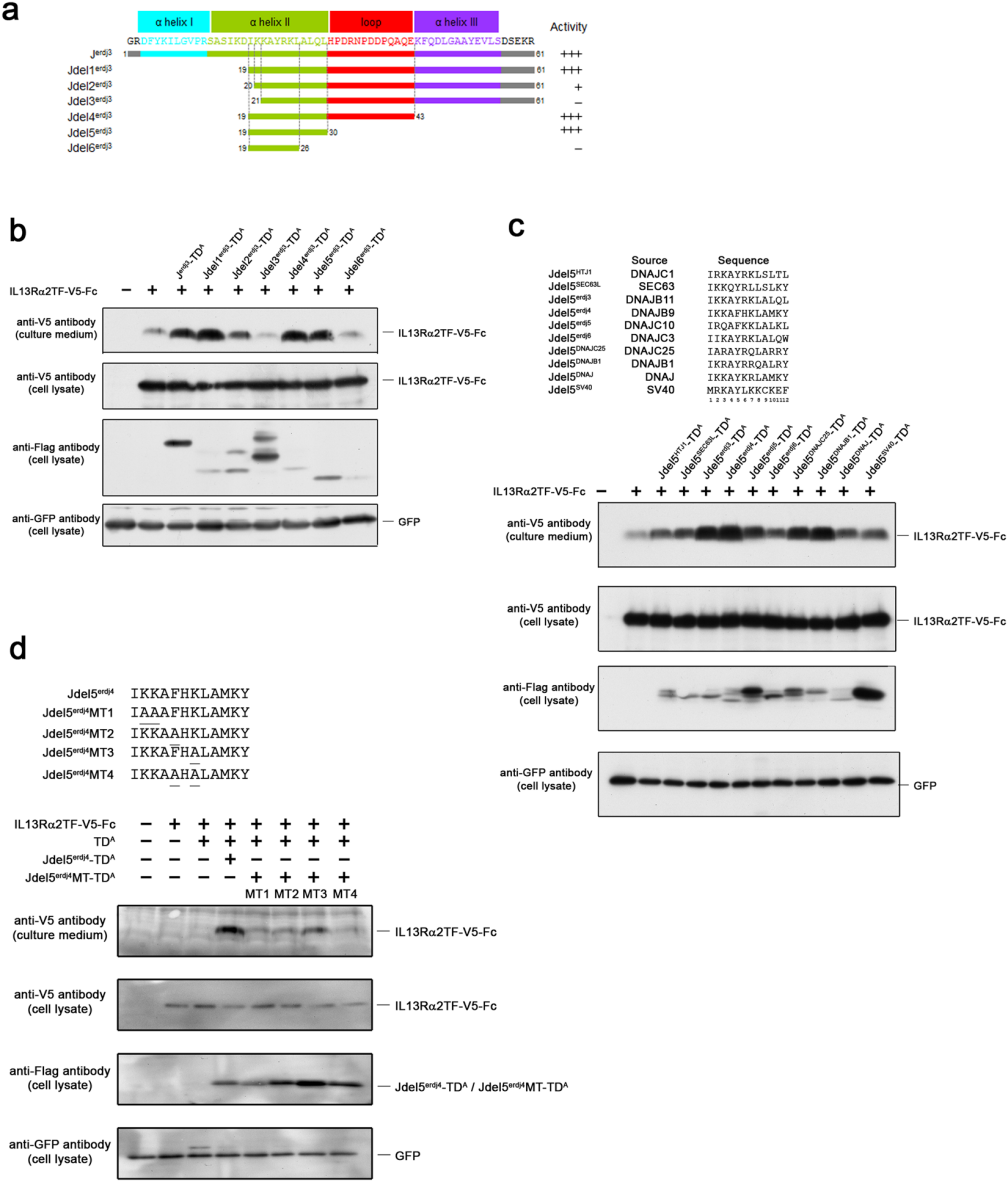
Identification of the essential sequence. (**a**) The alpha helixes and loop domains of the J-domain fragment sequence are indicated by brackets. As shown in the alignment, the original J-domain fragment sequence is 61 amino acids in length. Truncated J-domain fragment sequences used in this assay were depicted (Jdel^erdj3^-Jdel6^erdj3^). (**b**) The original sequence (J^erdj3^) and deletion polypeptides derived from a Erdj3 J-domain fragment sequence (Jdel^erdj3^ ~ Jdel6^erdj3^) were linked to protein A, forming a fusion protein that was co-expressed with a V5-tagged IL13Rα2-Fc target protein in transfected cells. The level of expression of the V5-tagged target protein in culture media was determined in an immunoblot assay using an anti-V5 antibody, and expression of fusion proteins carrying an original or truncated J-domain sequence was detected in cell lysate by western blot analysis using an anti-Flag antibody. All transfectants were co-transfected with a reporter plasmid expressing GFP. The cell lysate membrane was subsequently probed with an anti-GFP antibody (as a transfection control) (bottom panel). (**c**) The corresponding sequences from various J-domain proteins are aligned. The corresponding sequence derived from other J-domain proteins was conjugated to protein A to make a fusion protein, which was co-expressed with a V5-tagged IL13Rα2-Fc target protein in transfected cells. The V5-tagged IL13Rα2-Fc target proteins in both culture media and cell lysates were analyzed by western blot analysis using an anti-V5 antibody, and the fusion proteins incorporating the corresponding sequences derived from DnaJ protein were detected in cell lysate with an anti-Flag antibody. The cell lysate membrane was subsequently probed with anti-GFP antibody as a transfection control (bottom panel). (**d**) For mutation analysis, the second and third amino acids in the corresponding sequence from Erdj4 (IKKAFHKLAMKY) were substituted for alanine and conjugated to protein A (MT1: IAAAFHKLAMKY). MT2 (IKKAAHKLAMKY) and MT3 (IKKAFHALAMKY) have a point mutation at 5^th^ and 7^th^ amino acid, respectively, and MT4 has both mutations at the same position (IKKAAHALAMKY). The fusion proteins with native or mutated sequence (Jdel5^erdj4^-TD^A^ or Jdel 5^erdj4^MT-TD^A^) were expressed with a V5-tagged IL13Rα2-Fc target protein in HEK293 cells. The protein levels of the V5-tagged IL13Rα2-Fc target protein in both transfected cells and culture media were analyzed by western blot analysis using an anti-V5 antibody. The protein-A fusion proteins in transfected cells were analyzed using an anti-Flag antibody. The cell lysate membrane was subsequently with anti-GFP antibody as a transfection control (bottom panel).

We performed a series of experiments in order to identify the essential amino acid sequence in the engineered J-domain fusion protein. We constructed a fusion protein with protein A as the targeting domain, conjugated to various deletion mutants of a J-domain fragment sequence, and V5-tagged IL13Rα2TF-Fc target protein was expressed with the fusion protein in HEK293 cells. The culture medium was harvested, and the secreted V5-tagged IL13Rα2TF-Fc target proteins were analyzed. Figure 5a depicts the domain configuration of a J-domain fragment sequence from Erdj3. The J-domain fragment sequence identified in the initial screening was composed of three alpha helices (helices I, II, and III) and one loop that connected helices II and III. As shown in the alignment, the original sequence is 61 amino acids in length, and a series of polypeptides were generated from N- and C-terminal deletion of the original sequence.

While the deletion of either the first alpha helix (helix I) or helix I plus part of helix II had comparable activities, the deletion of additional amino acids after 19^th^Ile abolished the activity (Fig. 5b, lane 5). In the loop sequence, the HPD motif has been widely conserved among J-domain proteins and it is well accepted that the HPD motif is essential for the functional J-protein–Hsp70 interactions. Even if the loop sequence was deleted from the C-terminus, however, the effect was still observed (del5). In contrast, the activity sharply dropped when an additional four amino acids were deleted from alpha helix II (del6; lane 9). As noted before, the difference in the activity of the del1 polypeptide and that of the del2 polypeptide suggested that the 19^th^Ile residue was essential for activity. Serial deletion mutants from the C-terminus sequence revealed that a tetra amino acid sequence from the 27^th^ Ala is also important for activity (del5 and del6). In this study, polypeptide del5 (IKKAYRKLALQL) defines the minimal polypeptide sequence for supporting complete activity. The identified sequence is located within α helix II of the J-domain fragment, but it does not include any residues of the adjacent (C-proximal) loop domain.

The corresponding sequences extracted from other J proteins were inserted into the fusion protein carrying protein A and expressed with V5-tagged IL13Rα2TF-Fc target protein in HEK293 cells to investigate whether these sequences also possessed activity. Although all tested sequences had some activity, the corresponding sequence from Erdj4 (Jdel5^erdj4^; IKKAFHKLAMKY) had remarkable activity (Fig. 5c, lane 6). Sequence alignment analysis implied a conserved basic amino acid at the 2^nd^ or 3^rd^ position and another basic amino acid at the 6^th^ or 7^th^ position. We substituted two Ala residues for 2^nd^ and 3^rd^ basic amino acids of Jdel5^erdj4^ (Jdel5^erdj4^MT; IAAAFHKLAMKY) and expressed this construct with V5-tagged IL13Rα2TF-Fc target protein in HEK293 cells. This substitution at the 2^nd^ and 3^rd^ position completely abolished the activity, suggesting this basic amino acid sequence is necessary (Fig. 5d). Another mutation at 5^th^ Phenylalanine and 7^th^ lysine also reduced the activity and the effect dropped sharply when both amino acids were substituted (Fig. 5d).

### Engineered J-domain fragment fusion protein for protein-folding diseases

Protein folding issues underlie many diseases, including cystic fibrosis, Alpha-1 antitrypsin deficiency, polyQ diseases, and Alzheimer’s disease^27^. For this reason, they are known as “conformational diseases.” Alpha-1 antitrypsin deficiency (AT deficiency) is an autosomal genetic disorder. AT deficiency occurs worldwide, affecting roughly 1 in 2,857 to 5,097 individuals with European ancestry^28^. AT is a 52-kDa serine protease inhibitor produced mainly in the liver. Over 90 genetic variants have been identified in the *protease inhibitor 1* (*PI*) gene on chromosome 14 that are associated with AT deficiency. The most common basis of AT deficiency is the Z mutation, a single base-pair substitution of Glu to Lys at codon 342 (Glu342Lys; ATZ). Homozygosity for the Z mutation typically result in emphysema in young adults and liver disease in certain infants and adults^29^. The ATZ protein cannot fold to establish a normal (M) conformation and consequently accumulates in aggregates within the ER. While abnormally folded ATZ has a slow secretion and significantly reduces AT levels in blood and body fluids, the ATZ that is secreted is functional^30^. Accumulation of aggregated ATZ in liver cells leads to liver toxicity and liver cirrhosis^31, 32^.

**Figure 6:**
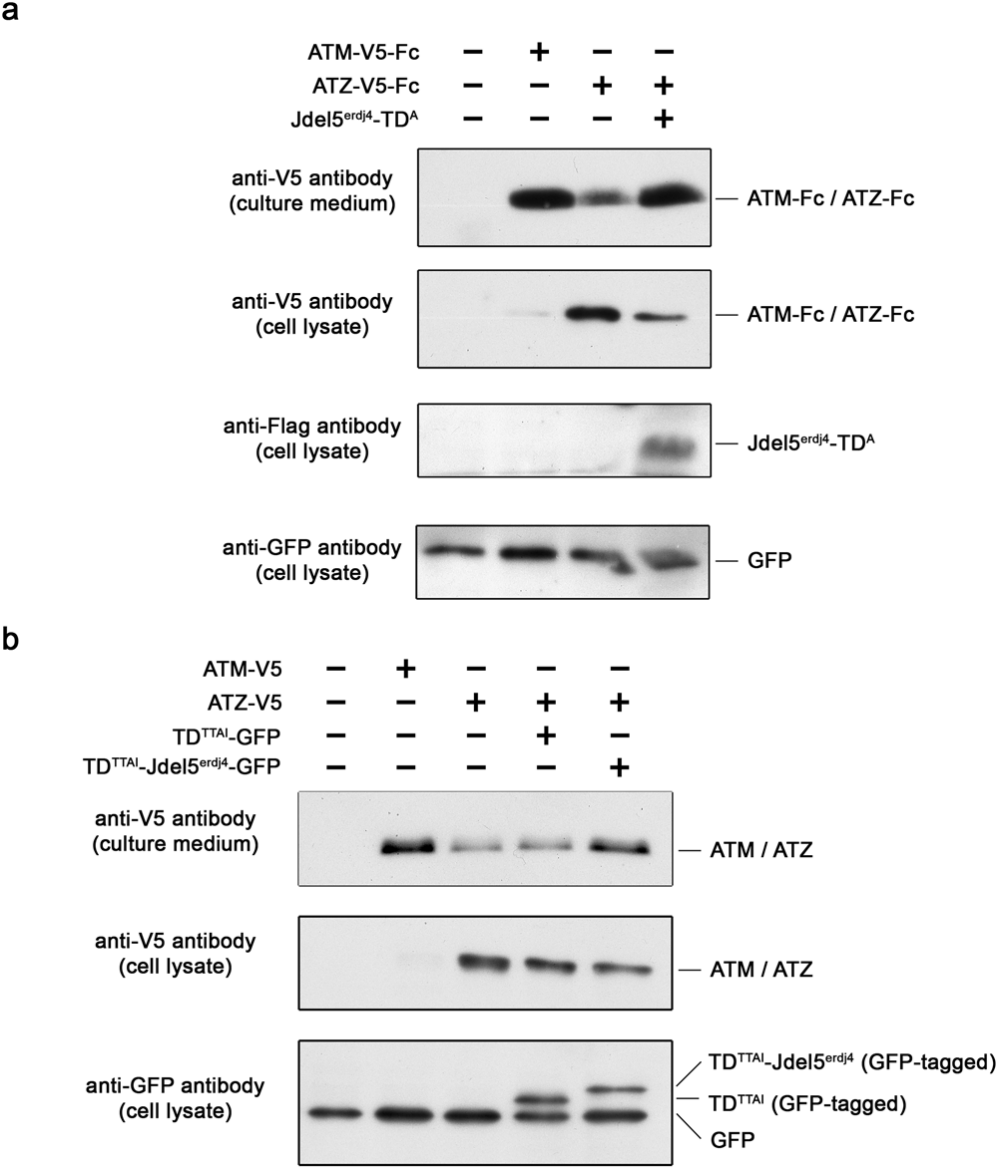
J-domain fragment fusion protein for α1 antitrypsin deficiency. (**a**) HEK293 cells were transfected to express Fc-fusion proteins of normal M form AT (ATM) or mutant type of AT (ATZ) with (+) or without (−) a Jdel5^erdj4^ fusion protein targeting Fc (Jdel5^erdj4^-TD^A^). Two days later, the cell culture medium (top panel) and cell lysate (second, third and bottom panels) were harvested, and ATM-Fc or ATZ-Fc was detected by western blot. ATM-Fc was efficiently secreted (lane 2), while ATZ-Fc was largely retained in cells (lane 3). The co-expression of the fusion protein of Jdel5^erdj4^ (Jdel5^erdj4^-TD^A^) significantly enhanced the secretion of ATZ-Fc and cleared ATZ-Fc from cells (lane 4). The control (left lane) indicates no transfection control. The cell lysate membrane was subsequently probed with an anti-GFP antibody as a transfection control (bottom panel). (**b**) Jdel5^erdj4^ sequence was conjugated to ATZ binding-tetrapeptide (TTAI). Tetrapeptide sequence without Jdel5^erdj4^ was used as a control. In order to ensure the expression of the small polypeptide, these polypeptide sequences were expressed as GFP fusion proteins (TTAI- Jdel5^erdj4^-GFP and TTAI-GFP). V5-tagged mutant type of AT (ATZ) was expressed with these polypeptide fusion proteins in HEK293 cells, and expressed ATZ and polypeptide fusion proteins were detected using an anti-V5 antibody or anti-GFP antibody, respectively. GFP without any polypeptide sequence was also expressed to monitor transfection efficiency.

We investigated whether ATZ could be secreted by exploiting a J-domain fragment fusion protein. We fused coding sequences of ATZ with the Fc domain from human IgG to force an association between the artificial J-domain protein and ATZ. We observed that the ATZ-Fc protein was poorly secreted compared to normal (M; ATM) ATM-Fc (Fig. 6a, lane 2 vs. lane 3) but was secreted efficiently when ATZ-Fc was expressed with the engineered J-domain fragment fusion protein, and importantly, intracellular levels of ATZ-Fc were significantly reduced (lane 4).

Chang *et al*. discovered that the tetrapeptide TTAI was the tight-binding ligand for ATZ^33^. We took advantage of the TTAI sequence to establish whether a J-domain fragment fusion protein harboring TTAI peptide could be developed for therapeutic applications. The TTAI sequence was linked to the identified J-domain fragment sequence (Jdel5^erdj4^) and the fusion protein (TD^TTAI^-Jdel5^erdj4^) was expressed with V5-tagged ATZ. As observed with the Fc-fusion format, the secretion of the expressed ATZ was exceptionally hampered (Fig. 6b, lane 3) while ATM was well secreted (lane 2). When ATZ was expressed with GFP-tagged TTAI, the secretion of the ATZ was slightly enhanced (lane 4). Interestingly, the J-domain fragment fusion protein with TTAI significantly improved the secretion of the ATZ and intracellular protein levels were slightly decreased (lane 5). These results support our hypothesis that it may be possible to develop an ATZ-specific artificial J-domain protein to facilitate protein folding and secretion of ATZ and rescue the ATZ phenotype.

## Discussion

The ER is an intracellular compartment where many proteins, such as membrane proteins, lysosomal proteins, and secreted proteins, are produced and processed. The ER is also well-known as the place where the strict cellular quality control system monitors protein-folding status, and confines premature or misfolded proteins. As a result of this strict system, many proteins are trapped in the ER and eventually degraded.

Misfolding or unfolding of proteins affects protein function, and for secreted proteins, confinement in the ER and degradation by the cellular quality control system directly affects protein yield. In fact, many efforts have been directed toward understanding and tailoring these systems for recombinant protein expression. We initially tried to control the chaperone and cellular quality control systems through general overexpression of the chaperone protein (Bip), or the co-chaperone proteins, but these attempts did not improve the secretion of the target protein (Supplemental Fig. 1).

Chemical chaperones are a group of low-molecular weight compounds with chaperone activity, and it is thought that they bind to the exposed hydrophobic surfaces of proteins and inhibit protein aggregation. Thus, chemical chaperones can help proteins to escape the cellular quality control system non-specifically^34^. We expressed Fc-fusion proteins in human cells and incubated the cells with 4-phenylbutyric acid (4-PBA), one of the most widely used chemical chaperones, but 4-PBA did not improve the secretion of Fc-fusion proteins (Supplemental Fig. 2).

In order to achieve more specific control of the expression of the target protein, we first conjugated domains derived from chaperone proteins and co-chaperone proteins to a target protein (IL13Rα2) that was easily degraded as a result of a protein-folding problem. Through screening, we identified a set of protein subdomains from the DnaJ protein family. Next, the target proteins were expressed with a fusion protein containing the identified sequence as an effector domain and a target protein-binding domain to recruit the identified sequence close to the vicinity of the target protein, still rendering the system specific to the target protein. This methodology successfully enhanced protein secretion for many target proteins, including antibodies. Importantly, monoclonal antibodies produced with this technique maintained their ability to bind to the corresponding epitope, suggesting that the antibodies produced by this methodology held the proper protein conformation and heavy/light chain arrangement.

The fragment sequences identified through screening contained helices I, II, and III of the J-domain. The J-domain family is widely conserved in species ranging from prokaryotes (DnaJ protein) to eukaryotes (Hsp40 protein family). All DnaJ family proteins are characterized by the presence of a canonical J-domain responsible for stimulating ATP hydrolysis in the Hsp70 protein nucleotide-binding domain (NBD), which drives conformational changes in the Hsp70 substrate-binding domain (SBD)^35^. The DnaJ protein family is categorized into four types (I, II, III, and IV) based on domain configuration. Type I J proteins consist of a glycine/phenylalanine-rich linker (G/F region), a zinc-finger domain, and a carboxy-terminal domain. Type II J proteins possess all these domains except for the zinc-finger domain, and type III J proteins only feature the J-domain positioned at its carboxy terminus. Type IV is a relatively recently discovered J protein family that has a J-domain-like domain. Considering the high structural divergence in these domain configurations, these extra domains could provide substrate specificity, spatial/temporal control, or additional functions. Contrary to the divergence in domain architecture across species, the J-domain structure has been highly conserved among all members. The J-domain is composed of four helices: I, II, III, and IV. Helices II and III are connected via a flexible loop containing an “HPD motif”. The HPD motif exists in all J proteins, except type IV J proteins; therefore, type IV J proteins are termed J-domain-like proteins.

In the composition of the J-domain, it is thought that the HPD motif is fundamental to primitive activity^36^. Contrary to our expectation, deletion of the HPD motif from the J-domain sequence still showed activity to enhance the secretion of the target protein (Fig. 5). The data was further reinforced by the mutation in the HPD motif (Supplemental Fig. 3). Within helix II, positively charged amino acids are well conserved among the J-domain family. In fact, the basic amino acids in helix II are vital for DnaJ function^37, 38^. Interestingly, perturbation of the basic amino acids in the J-domain fragment sequence significantly decreases the activity (Fig. 5). The amino acid sequence in helix II conserved among the J-domain proteins, such as 5^th^ aromatic amino acid and basic amino acid positioned at 6^th^ or 7^th^ amino acid, also turned out to be important for activity (Fig. 5). Based on the sequence information obtained from the study, we performed a pull-down assay to investigate the interaction. We confirmed the ternary complex of Bip, the J-domain fragment fusion protein, and the target protein. Although interaction differences were expected between Bip and the target protein in the presence of the J-domain fragment fusion protein, a change in the interaction between Bip and the target protein was not detected (Supplemental Fig. 4).

To understand whether the forced interaction between the Bip protein and a target protein improves the secretion of the target protein, IL13Rα2TF-Fc was expressed with the fusion protein of Bip and protein A, though this failed to improve the secretion of IL13Rα2TF-Fc (Supplemental Fig. 5), suggesting that the simple recruitment of Bip protein to a target protein does not improve the secretion of the target protein.

As of yet, these data support that the identified J-domain fragment fusion protein works without Hsp70. Therefore, it is important to find the binding partner for the J-domain fragment sequence. Lau *et al*. developed a series of peptides for the selective capture of aggregated prion protein (PrP) from plasma and later they revealed that the selected peptide also binds to aggregated Aβ^39, 40^. They showed that positively charged amino acids in the peptide play an important role in the interaction with aggregated Aβ. Since the net charge of the identified J-domain fragment sequence is also positive and these basic amino acids are essential for its function, it is still conceivable that the identified J-domain fragment sequence works alone and binds to a misfolded protein. An *in vitro* binding assay or *in vitro* folding assay reconstituted by purified proteins would be useful to clarify the detailed molecular mechanism of the J-domain fragment fusion protein.

In Fig. 6, the Fc-fusion protein with ATZ (ATZ-Fc) was constructed to induce an association between ATZ-Fc and the artificial J-domain fragment protein carrying protein A. We observed that ATZ-Fc was poorly secreted compared to normal AT (ATM) (Fig. 6a, lane 2 vs. lane 3), but it was secreted efficiently when co-expressed with the J-domain fragment fusion protein (lane 4). We further developed the system for AT deficiency by exploiting the tetrapeptide TTAI to target ATZ, which demonstrated great potential for improving the disease phenotype. Further engineering of the target protein binding sequence will assure its prominent effect in therapeutic applications, and develop other disease-specific J-domain fragment fusion proteins to rescue other conformational disease phenotypes, such as that of cystic fibrosis.

## Materials and Methods

### Cells and antibodies

HEK-293 cells (human embryonic kidney cells) and the plasmid-expressing humanized anti-IL-8 antibody (p6G425V11N35A.choSD; catalogue No. 209552) were purchased from the American Type Culture Collection (Manassas, VA). V5 and Flag monoclonal antibodies, anti-GST antibody, rabbit anti-GFP antibody, horseradish peroxidase (HRP)-conjugated mouse IgG antibody, and HRP-conjugated rabbit IgG antibody were purchased from GenScripts (Piscataway, NJ). HRP-conjugated antibody against human IgG1 was purchased from EMD Millipore (Darmstadt, Germany).

### Plasmid preparation

#### Backbone plasmid construction

pcDNA3 (Life Technologies, Grand Island, NY) with a modified multiple cloning site (MCS) was used as a backbone plasmid. DNA molecules encoding protein sequences were obtained by polymerase chain reaction (PCR), gene synthesis, or the annealing of complementary DNA molecules with standard protocols. DNA molecules encoding the amino acid sequence of an immunoglobulin Fc region (IgG_1_), human high affinity immunoglobulin gamma Fc receptor I (GenBank: NM_000566.3), macacine gammaherpesvirus 5 R1 (GenBank: NC_003401.1), staphylococcal protein A (GenBank: X70422.1), streptococcus G148 protein G (GenBank: X53324.1), human alphaherpesvirus 1 envelope glycoprotein E (GenBank: NC_001806.2), and human herpesvirus 1 glycoprotein I (GenBank: GQ898901.1) were produced by standard gene synthesis. A DNA molecule having a DNA sequence, GGAGGCGGAAGTGGTGGGAGCGGTGGAAGCGGAGGC, encoding the glycine-serine linker sequence, GGGSGGSGGSGG, was obtained by annealing complementary single strands synthesized by standard methods. The cDNA sequence for the signal sequence from human insulin (MALWMRLLPLLALLALWGPDPAAA) was annealed and inserted into the backbone plasmid.

DNA sequences encoding the IL13Rα2 protein, truncated IL13Rα2 protein (amino acids 1–339), or truncated TNFRSF1A (TNFRSF1ATF; aa 1–211) protein were inserted into the plasmid. DNA sequences encoding truncated TNFRSF1B (TNFRSF1BTF; aa 1–257) were amplified as a Fc fusion gene by a standard PCR method.

To produce fusion proteins comprising a J-domain fragment sequence, DNA molecules encoding J-domain fragment sequences were sub-cloned into the plasmid to yield a J-domain fragment-Flag-pcDNA plasmid vector. DNA molecules encoding human signal sequence, the glycine-serine linker, and targeting domain (TD) were inserted into the vector in frame.

J-domain sequences used in the screening are as follows:

J^DNAJB1^: MGKDYYQTLGLARGASDEEIKRAYRRQALRYHPDKNKEPGAEEKFKEIAEAYDVLSDPRKREIFDRY (67 aa)

J^DNAJC5^: MADQRQRSLSTSGESLYHVLGLDKNATSDDIKKSYRKLALKYHPDKNPDNPEAADKFKEINNAHAILTDATKRNIYDKYGSLGLYV (86 aa)

J^SV40^: MDKVLNREESLQLMDLLGLERSAWGNIPLMRKAYLKKCKEFHPDKGGDEEKMKKMNTLYKKMEDGVKYAHQPDFGGFWDA (80 aa)

J^erdj3^: GRDFYKILGVPRSASIKDIKKAYRKLALQLHPDRNPDDPQAQEKFQDLGAAYEVLSDSEKR (61 aa).

### Expression and detection of proteins in HEK293 cells

Expression vector plasmids encoding various protein constructs were transfected into HEK293 cells with FuGENE transfection reagent (catalog no. E2311, Promega). As indicated in the following examples, a separate plasmid expressing green fluorescent protein (GFP) was co-transfected with each expression vector plasmid encoding a target protein and/or a fusion protein to monitor transfection efficiency. Cultures of transfectant cells were incubated for two days. Culture medium and/or cell lysates were analyzed for expressed proteins using western immunoblot assays. Samples of culture media were centrifuged to remove debris prior to analysis. Cells were lysed in a lysis buffer (10 mM Tris-HCl, pH 8.0, 150 mM NaCl, 10 mM EDTA, 2% SDS) containing 2 mM PMSF and protease cocktail (cOmplete Protease Inhibitor Cocktail; Sigma). After brief sonication, the samples were analyzed for expressed proteins using western immunoblot assays. For western blot analysis, samples were boiled in an SDS-sample buffer and run on polyacrylamide electrophoresis. Thereafter, the separated protein bands were transferred to a PVDF membrane.

The expression of GFP as an internal transfection control was detected using anti-GFP antibodies. Expressed proteins in western blots were detected using a chemiluminescent signal. Briefly, blots were reacted with a primary antibody capable of binding the particular epitope tag (e.g., V5 or FLAG) carried by the proteins. After rinsing away the unreacted primary antibody, a secondary, enzyme-linked antibody (e.g., HRP-linked anti-IgG antibody) was allowed to react with the primary antibody molecules bound to the blots. Following rinsing, a chemiluminescent reagent was added, and the resultant chemiluminescent signals in the blots were captured on X-ray film.

### ELISA (enzyme-linked immunosorbent assay)

The activities of the proteins of interest were evaluated using standard assay techniques. Briefly, to monitor the activity of monoclonal antibodies, an enzyme-linked immunosorbent assay (ELISA) was conducted with a standard protocol. GST-fused recombinant human IL-8 or TNFα was expressed and purified from *E. coli* as an epitope for DP12 (anti-IL8 antibody), truncated TNFRSF1B (TNFRSF1BTF), or truncated TNFRSF1A (TNFRSF1ATF), respectively. Microtiter plate wells were coated overnight at 4 °C, and after coating, the wells were washed three times with TBST (20 mM Tris-HCl, pH 8.0, 150 mM NaCl, 0.05% Tween 20), blocked for one hour with TBST containing 5% nonfat milk, and washed again with TBST. Samples harvested from the culture medium were added to the well and incubated for one hour at room temperature. The wells were then washed three times with TBST, incubated with HRP-linked anti-human IgG antibody in TBST containing 3% BSA for one hour, washed three times with TBST, and washed again three times with TBST. Finally, peroxidase activity was assayed with 0.01 mg/ml 3′,3′,5′,5′-tetramethylbenzidine (TMB) and 0.01% H_2_O_2_ in a buffer of 0.1 M sodium acetate (pH 6.0). After an equal volume of 1 M HCl was added, the optical density was determined at 450 nm.

## Electronic supplementary material

Supplementary Dataset
