# Supplementary material for: Artificial DnaJ Protein for protein production and conformational diseases
Source: Sci Rep. 2017 Aug 17;7:8531. doi: 10.1038/s41598-017-09067-7 (PMC5561034; doi:10.1038/s41598-017-09067-7)
Supplement: Supplementary file 1 — Supplementary Dataset [file 41598_2017_9067_MOESM1_ESM.doc]

**Artificial DnaJ Protein for protein production and conformational diseases**

Akinori Hishiya*, and Keizo Koya

Sola Biosciences, Inc.,

27 Strathmore Road, Natick, MA 01760, USA

*akinori.hishiya@sola-bio.com

**Supplemental Data**


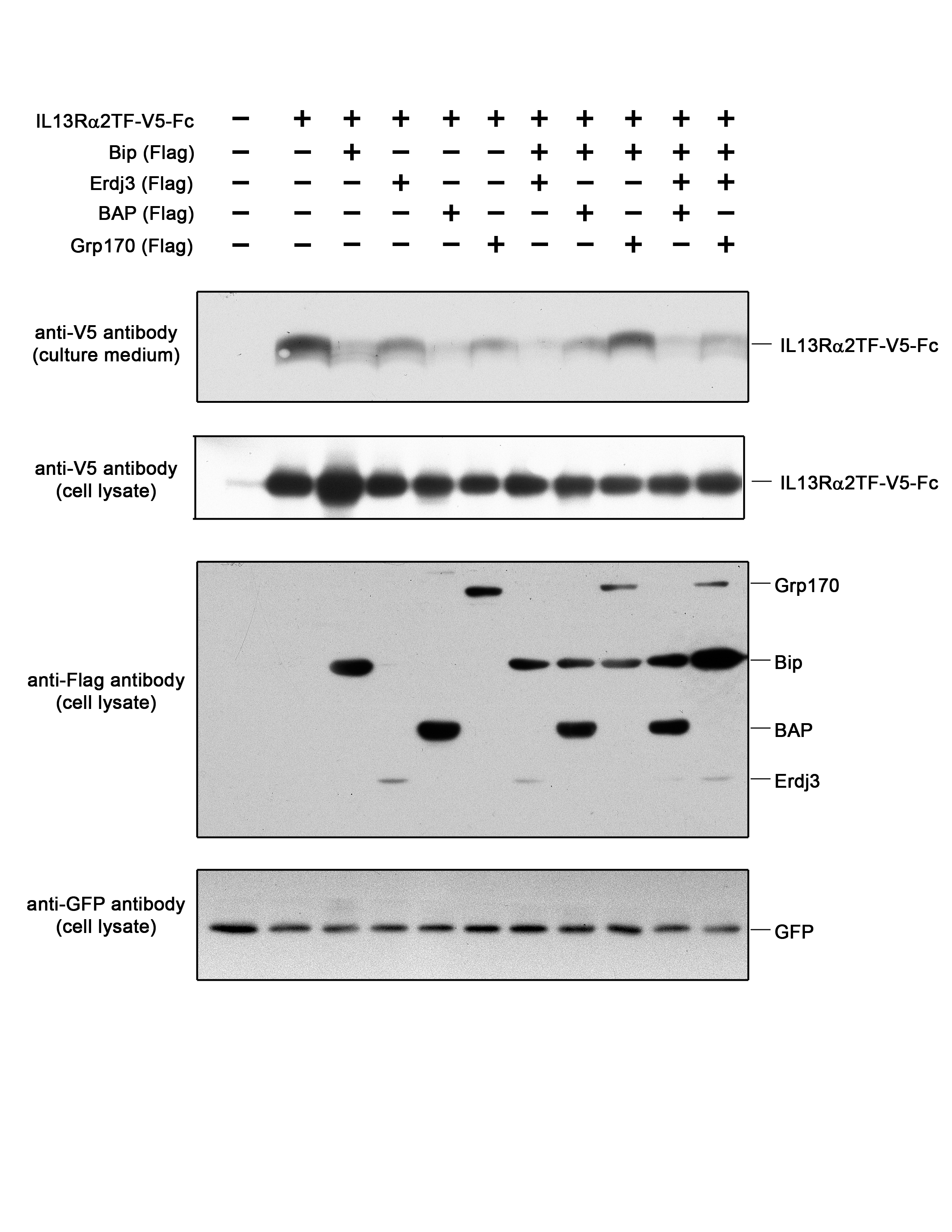


**Supplemental Figure 1**

IL13Rα2TF-V5-Fc was expressed in the presence (“+”) or in the absence (“-”) of various proteins, such as chaperone/co-chaperone proteins in HEK293 cells. Cells were cultured for 48 hours after transfection and the expressed IL13Rα2TF-V5-Fc was detected by western blot analysis in the culture media (top panel) and in the cell lysate (second panel). Reporter GFP was also expressed and monitored as a transfection control (bottom panel).


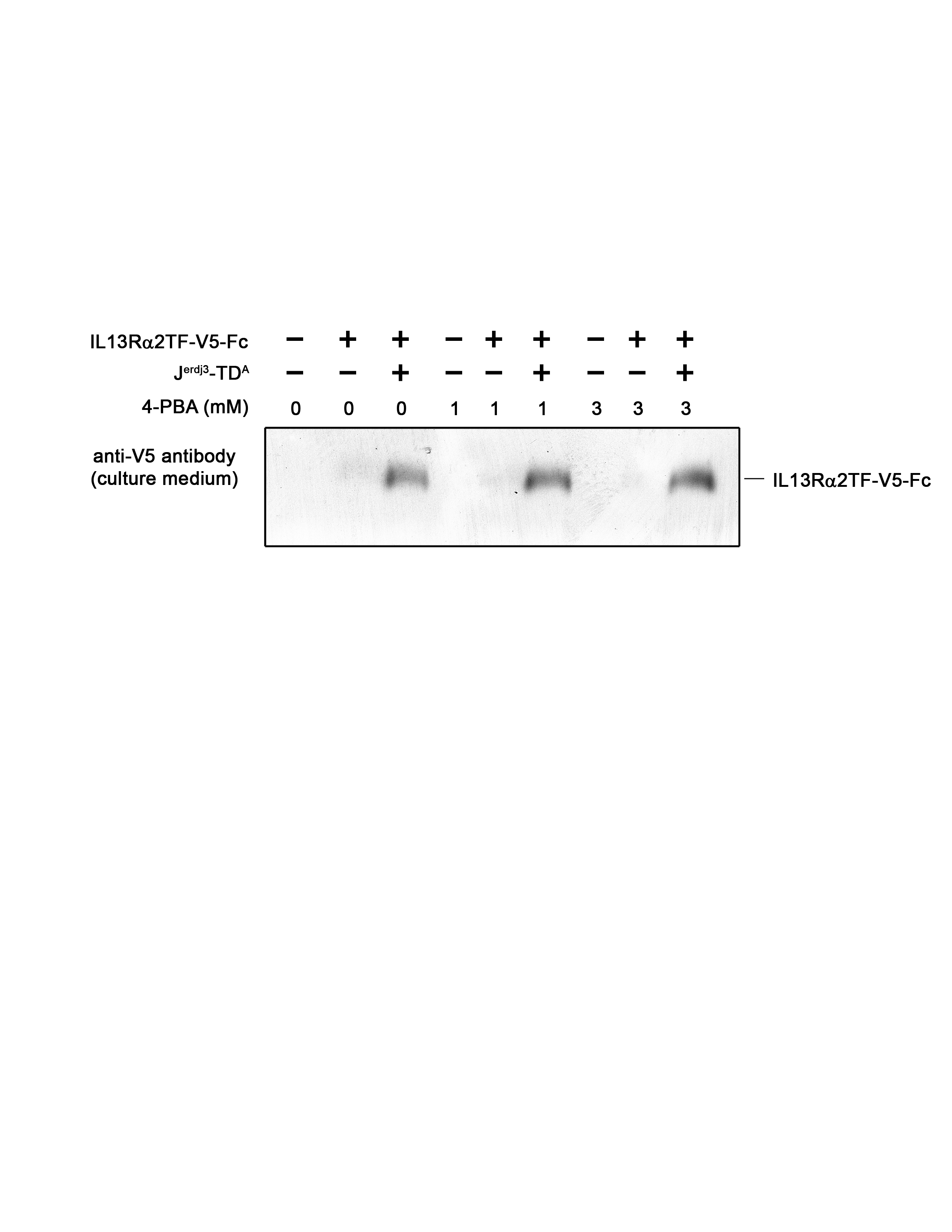


**Supplemental Figure 2**

IL13Rα2TF-V5-Fc was expressed in HEK293 cells with (+) or without (–) a J domain fragment fusion protein incorporating protein A (Jd5^erdj4^-TD^A^; lane 4). 24 hours later, the cell culture medium was changed with a fresh medium including 1mM 4-phenylbutyrate (4-PBA) (lane 4-6), or 3mM 4-PBA (lane 7-9), and the cells were incubated for another 24 hours. The culture medium was harvested and the production of IL13Rα2TF-V5-Fc was analyzed by western blot assay using an anti-V5 antibody.


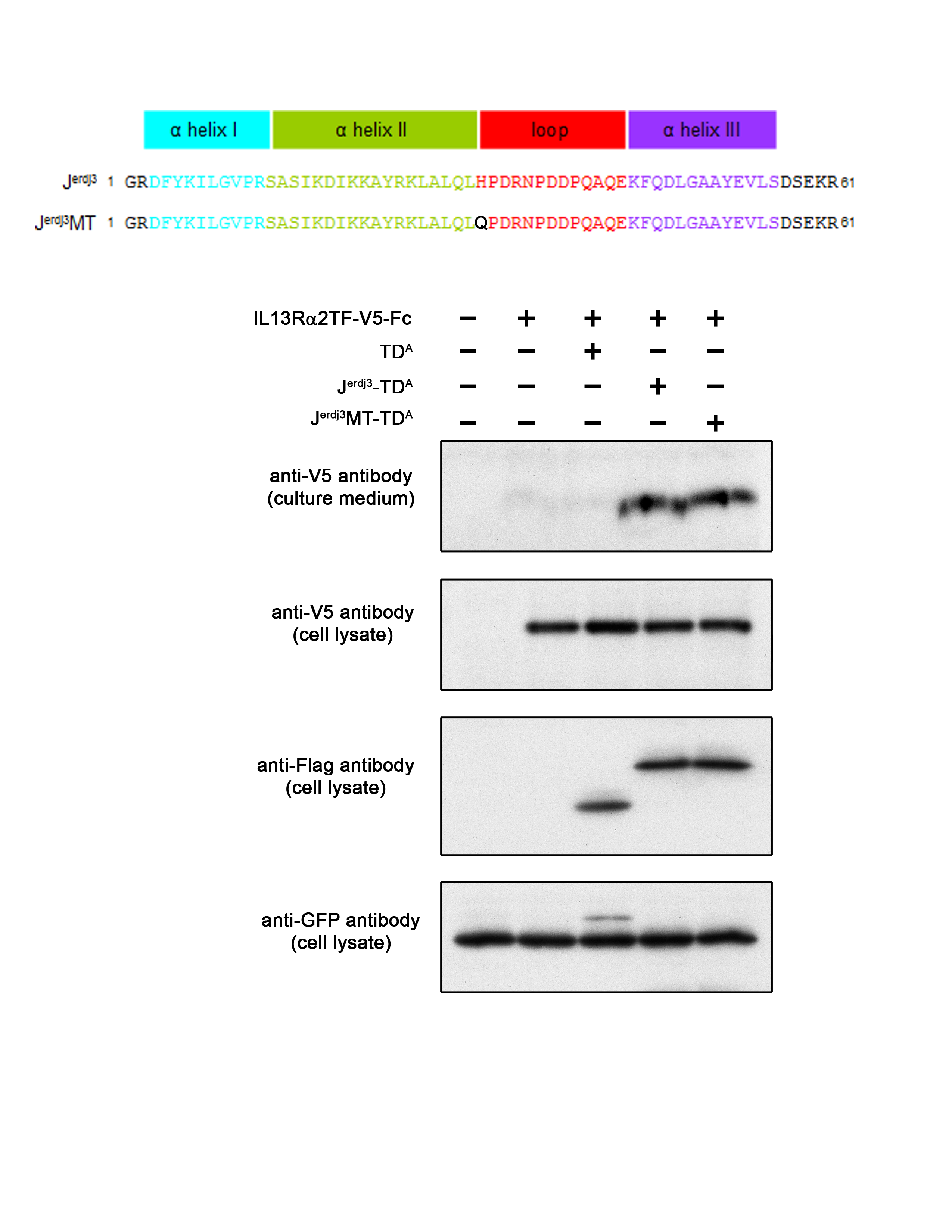


**Supplemental Figure 3**

J-domain fusion protein carrying a mutation in HPD sequence was expressed with Fc-fusion proteins of IL13Rα2TF (IL13Rα2TF -Fc) in HEK293 cells. Two days later, the cell culture medium (top panel) and cell lysate (bottom panel) were harvested, and IL13Rα2TF -Fc was detected by western blot using an anti-V5 antibody.


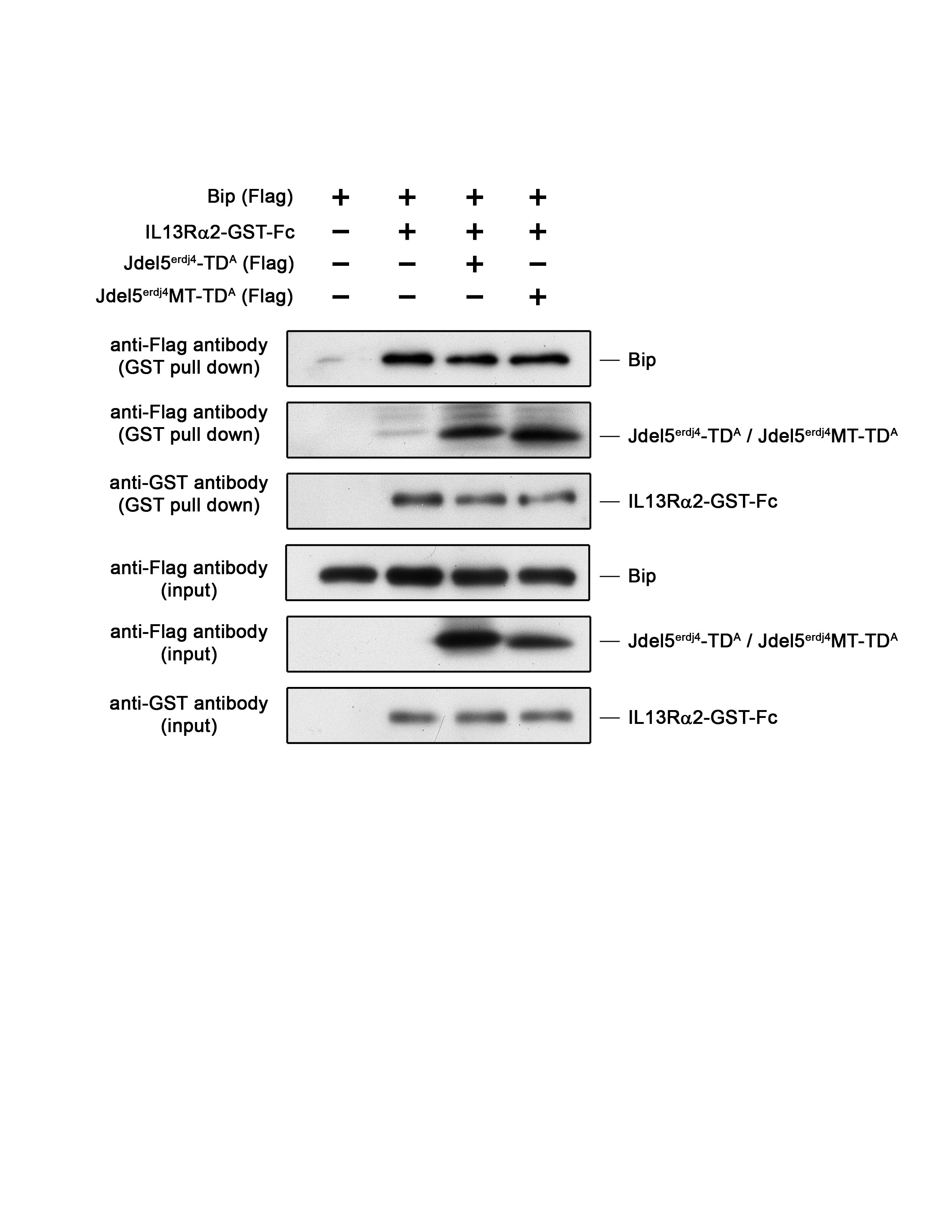


**Supplemental Figure 4**

The Fc fusion protein with GST-tagged IL13Rα2TF (IL13Rα2TF-GST-Fc) was expressed in HEK293 cells with (+) or without (–) Flag-tagged Bip and Flag-tagged J domain fragment fusion protein incorporating protein A (Jdel5^erdj4^-TD^A^; lane 3) or mutant type of J domain fragment (Jdel5^erdj4^MT-TD^A^; lane 4). 24 hours later, the cell culture medium was replaced with a fresh medium including 0.1µM bafilomycin A1 (BFA) to inhibit vesicle transportation, and the cells were cultured for another 24 hours. The cells were lysed in pull-down assay buffer (20 mM Tris-HCl, pH 7.5, 150 mM NaCl, 1 mM EDTA, 1 mM EGTA, protease inhibitor cocktail, 1% Triton X-100), and after brief sonication, the cell debris was removed through centrifugation. The supernatant was then incubated with GSH beads for two hours. After the beads were washed with pull-down assay buffer, the complex was dissolved on SDS-PAGE. The top, second and third panels show Flag-tagged Bip protein, Flag-tagged J domain fragment fusion protein, and IL13Rα2TF-GST-Fc proteins precipitated with IL13Rα2TF-GST-Fc proteins, respectively. The expression of Flag-tagged Bip protein, Flag-tagged Jdel5^erdj4^/Jdel5^erdj4^MT fusion protein, and IL13Rα2TF-GST-Fc, is shown in the fourth, fifth and bottom panel, respectively.


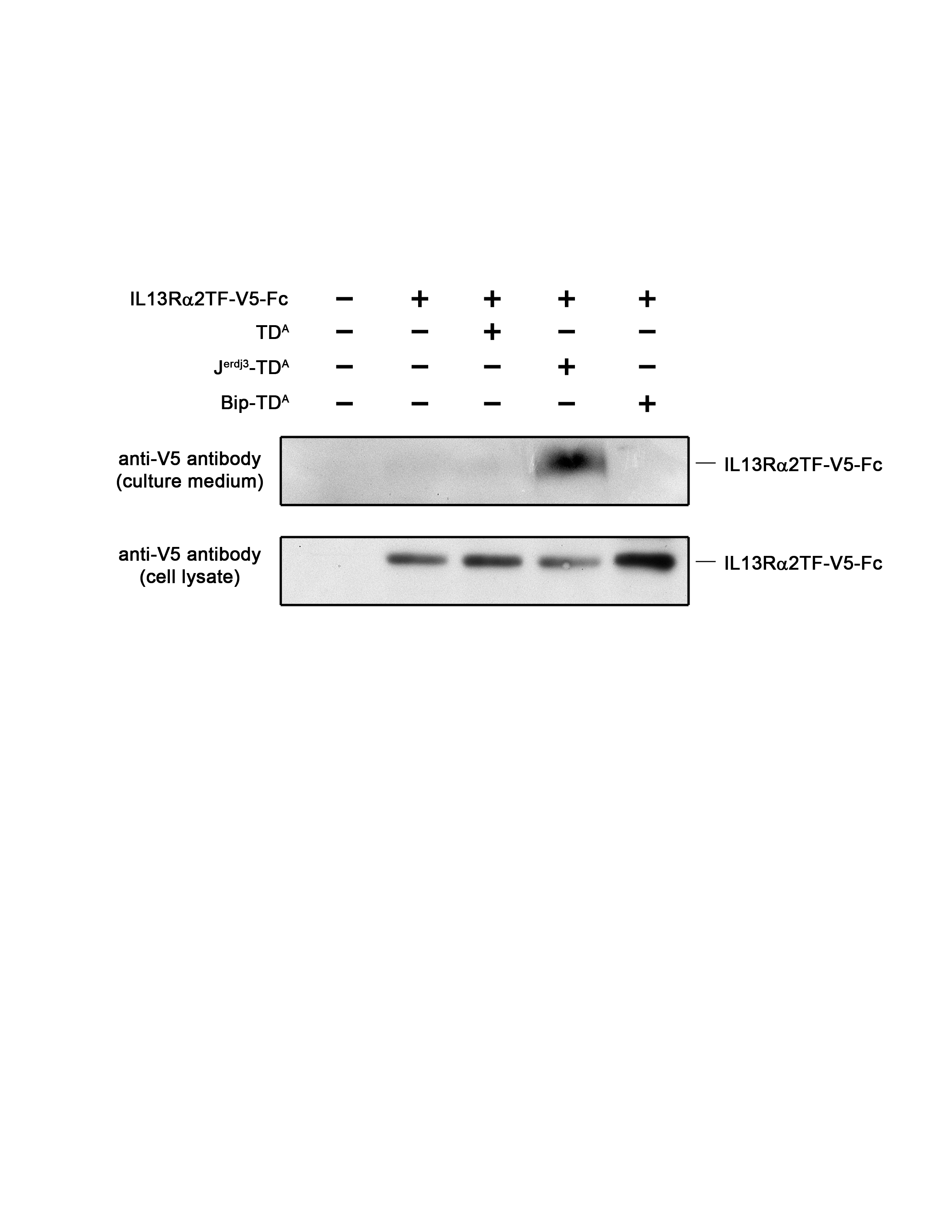


**Supplemental Figure 5**

HEK293 cells were transfected to express Fc-fusion proteins of IL13Rα2TF (IL13Rα2TF -Fc) with (+) or without (–) a target domain only (TD^A^; lane 3); a J domain fragment fusion protein (Jdel5^erdj4^-TD^A^; lane 4); or a fusion protein in which Bip protein is linked to protein A (Bip-TD^A^; lane 5). Two days later, the cell culture medium (top panel) and cell lysate (bottom panel) were harvested, and IL13Rα2TF -Fc was detected by western blot using an anti-V5 antibody.
